# Seeing Through Christ's Eyes

**DOI:** 10.1097/CNJ.0000000000001302

**Published:** 2026-06-08

**Authors:** Gabriela Marquez, Adrianna Watson

**Affiliations:** **Gabriela (Gaby) Marquez** is a senior nursing student at Brigham Young University. Gaby also serves as a research assistant for Dr. Adrianna Watson and is passionate about fostering love and light in the nurse–patient healing relationship.; **Adrianna Watson, PhD, RN, CCRN, TCRN,** is an assistant professor and nurse researcher at Brigham Young University where she mentors undergraduate nursing students in research and clinical practice.

During one of my pediatric rotations in my nursing program, I learned a lesson in humility and the importance of choosing to not judge others. This experience profoundly shaped how I see myself and the kind of nurse I want to become.

It started with a hectic day in the pediatric unit with its overflow of med-surg patients. My preceptor and I were monitoring and caring for two patients, one of whom was an infant. The infant's mother had been staying in the room with the baby for several days and was visibly exhausted.

During morning rounds, I noticed the mother's withdrawn demeanor. Her exhaustion made her seem distant. My preceptor's comments about her did not help; she described the mother negatively, which in turn influenced my perception. I found myself withdrawing, too, not interacting as warmly and openly with the mom as I usually would. Looking back, I feel frustrated I let this happen because my favorite part of nursing is connecting with patients and families to build a relationship of trust that can transform their hospital experience.

The turning point came later that day when I spoke with the infant's mother one on one. I had an assignment to complete a spiritual assessment, and I approached her for permission to conduct the interview. She agreed, and what began as a simple conversation turned into an hour-long exchange that deeply impacted me.

The mother shared her story. Her 6-month-old baby boy had undergone numerous surgeries. She was overwhelmed by the emotional and physical toll on her family. She revealed her guilt about leaving her other children at home and being unable to spend more time with her husband. She admitted to moments of doubt, wondering whether the medical interventions were worth it or were causing her son unnecessary suffering. Despite her struggles, she expressed that her faith in Christ had given her strength and helped her see her son's journey as part of his earthly purpose.

As she opened up to me, I felt an indescribable love—a Christ-like love for her that brought me to the verge of tears. It was a privilege to feel that love because it gave me insight into how to care for her and her son honestly. In that moment, I regretted the judgments I had made earlier. Her exhaustion and quiet demeanor were not signs of rudeness but reflections of her immense burden.

That day, I learned a simple yet powerful lesson: Make connections with your patients. This mother expressed gratitude for the nurses who had taken the time to get to know her and be a source of strength when her family couldn't be there. Her words reinforced my commitment to building trust and relationships with those I care for, no matter how demanding the job may be.

Every time I go into clinical, I say a quick prayer, asking my heavenly Father to help me be the person my patients need. That interaction with the infant's mother is something I will never forget. It reminded me to see my patients and their families as God sees them. Although nursing can be emotionally and physically exhausting, I know that even small acts of kindness and understanding can profoundly impact someone's hospital experience.

Reflecting on this experience, I realized how easily I let someone else's perspective influence me. I'm grateful for the mother's patience and willingness to share her story, allowing me to learn from her. As a child, I was shy and anxious, often struggling to make friends. But those who showed kindness and helped me feel seen made all the difference. Their actions inspired me to be someone who brings comfort and connection to others. I honed these skills during mission service for my church, and now I'm learning to apply them as a nurse.

This experience reaffirmed my passion for nursing and my belief in the healing power of connection. Knowing how to help people feel loved, seen, and cared for is one of the greatest joys of this profession. I will carry the lessons this mother taught me throughout my nursing journey, striving to look beyond the surface and serve with compassion and humility.

